# Comparison of Electronic Health Record Messages to Mental Health Care Professionals Before vs After COVID-19 Pandemic

**DOI:** 10.1001/jamanetworkopen.2023.25202

**Published:** 2023-07-24

**Authors:** Simone A. Bernstein, Alison L. Huckenpahler, Ginger E. Nicol, Jessica A. Gold

**Affiliations:** 1 Department of Psychiatry, Washington University School of Medicine in St Louis, St Louis, Missouri

## Abstract

This qualitative improvement study examines the change in electronic health record messaging from patients to mental health professionals before vs after the COVID-19 pandemic.

## Introduction

The COVID-19 pandemic led to a global mental health crisis,^1^ with increased mental health–related visits across specialties and emergency departments.^2,3^ This study aims to quantify this increase by comparing prepandemic and postpandemic patient messaging in psychiatry. With inbox electronic health records (EHRs) volume as the main outcome, we hypothesized an increase in postpandemic messages.

## Methods

The Washington University in St Louis Institutional Review Board approved this qualitative improvement study. We followed the SQUIRE reporting guideline. This study analyzed EHRs (Epic Systems) of outpatients receiving psychiatric treatment at a large Midwestern academic medical center. We reviewed patient-initiated prepandemic (June 2, 2018, to March 18, 2020) and postpandemic (March 19, 2020, to January 3, 2022) messages for patients with at least 1 scheduled appointment in either period. Trends were assessed through September 2022 to observe whether increases persisted in early (April 1, 2020, to January 31, 2021) and late (November 1, 2021, to September 30, 2022) pandemic periods.

Message volume by patient sex and self-identified race (White and other [Black, Asian, and other or not specified]) was reviewed to understand population characteristics (eMethods in Supplement 1). Heteroscedastic *t* tests compared prepandemic and postpandemic outcomes of interest. Statistical significance was defined as *P* < .05.

## Results

Among 4724 patients (1636 males [34.6%]; mean [range] age, 35.4 [20.3-95.1] years; 135 Asian [2.9%], 644 Black [13.6%], 3807 White [80.6%], and 138 other or not specified [2.9%]), sending messages increased from 765 prepandemic to 4481 postpandemic messages (485.8%). Monthly message volume increased from 4661 prepandemic to 44 929 postpandemic messages (861.5%; per-patient mean [SD], 0.2 [0.1] vs 1.2 [0.4] messages; *P* < .001) (Figure, A). Among 174 professionals, there were 118 psychiatrists (67.8%), 15 social workers (8.6%), 13 therapists (7.5%), 13 psychiatric advanced practice clinicians, 7 psychologists (4.0%), 7 additional resource staff, and 1 case manager (0.6%). The mean number of professionals increased from 64.1 to 75.8 individuals (18.3%; *P* < .001).

**Figure.**
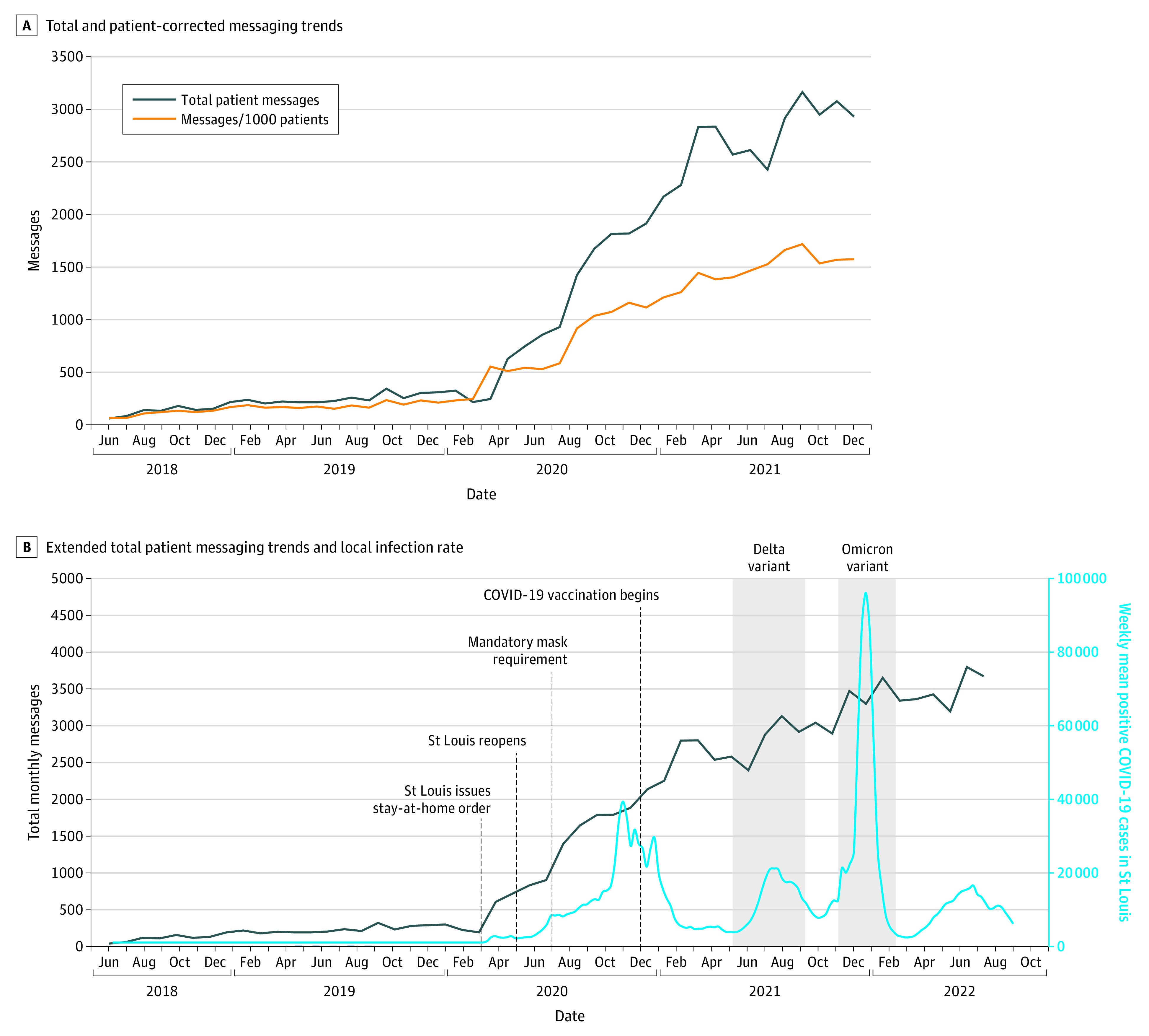
Messaging During the COVID-19 Pandemic During COVID-19, there was an increase in total messages sent and messages sent per patient. Additional observations in the extended study timeline show that message volume continued to increase independently of COVID-19 infection rates.

Trends were assessed through September 2022 to observe whether increases persisted in periods of lower COVID-19 infection rates (Figure, B).^4^ The mean (SD) number of messages per month was significantly lower in the early (1398 [560] messages) vs late (3414 [273] messages) pandemic (*P* < .001).

Males sent a higher mean (SD) number of prepandemic messages than females (7.5 [11.0] vs 5.4 [7.4] messages; *P* < .001) but fewer postpandemic messages (9.4 [13.7] vs 10.4 [16.3] messages; *P* = .03). Patients with other races sent a lower mean number of messages than White patients before (4.1 [4.4] vs 6.4 [9.5] messages; *P* < .001) and during (8.8 [12.5] vs 10.3 [16.0] messages; *P* < .001) the pandemic.

Patients messaged to ask medication questions (55.7%), general medical questions (40.4%), or their history (3.5%); cancel or request an appointment (0.2%); or request refills (0.2%). Patients primarily used the internet to send messages (60.7%), followed by the MyChart application (iOS: 28.1%; Android: 11.2%). Mean (SD; maximum) message length was 192 (234; 6651) characters.

## Discussion

Demand for mental health care increased profoundly during the pandemic, worsening patient access to mental health professionals. To our knowledge, this quality improvement study is the first to examine messaging volume in mental health care professionals. We report increased mental health service demand and increased messaging during the pandemic. This is consistent with observations of other specialty trends at another institution.^5^ The increase persisted well past the acute phase of the pandemic through subsequent infection surges.^4^ Increased messaging burden in this study reflects the sustained increase in patient need, which likely extends beyond psychiatry.

Increased messaging may also be associated with patient satisfaction, given that it is associated with message response time and more messages take more time.^6^ Patients may additionally have experienced disproportionate outcomes by race given that those identifying as White were more likely to send messages. This may have contributed to disparities in health care professional access.

This study has limited generalization to all clinical settings, including other specialties within our hospital. It included no analysis of message content, telephone calls, emails, or other patient–health care professional communication.

The pandemic led to a mental health crisis, which the messaging burden further demonstrates. Understanding patient-centric barriers to health care professional access and communication is critical to driving health system changes to improved patient care quality beyond the pandemic.

## Notes

Supplement 1. **eMethods.** Race Demographics

Supplement 2.
Data Sharing Statement
