# Supplementary material for: Comparison of Electronic Health Record Messages to Mental Health Care Professionals Before vs After COVID-19 Pandemic
Source: JAMA Netw Open. 2023 Jul 24;6(7):e2325202. doi: 10.1001/jamanetworkopen.2023.25202 (PMC10366699; doi:10.1001/jamanetworkopen.2023.25202)
Supplement: Supplement 1. — eMethods. Race Demographics [file jamanetwopen-e2325202-s001.pdf]

Supplemental Online Content

Bernstein SA, Huckenpahler AL, Nicol GE, Gold JA. Comparison of electronic health record messages to mental health care professionals before vs after COVID-19 pandemic. *JAMA Netw Open.* 2023;6(7):e2325202. doi:10.1001/jamanetworkopen.2023.25202

eMethods. Race Demographics

This supplemental material has been provided by the authors to give readers additional information about their work.

© 2023 Bernstein SA et al. *JAMA Network Open.*

eMethods. Race Demographics

Race was self-identified, and available categories were Asian, Black, White, and other or not specified. Because White patients were the overwhelming majority, data were analyzed into White and other partially for statistical significance for other groups, but also to reflect well-documented effects of minority status in the health care popuation.

© 2023 Bernstein SA et al. *JAMA Network Open.*
