# Supplementary material for: Comparison of Electronic Health Record Messages to Mental Health Care Professionals Before vs After COVID-19 Pandemic
Source: JAMA Netw Open. 2023 Jul 24;6(7):e2325202. doi: 10.1001/jamanetworkopen.2023.25202 (PMC10366699; doi:10.1001/jamanetworkopen.2023.25202)
Supplement: Supplement 2. — Data Sharing Statement [file jamanetwopen-e2325202-s002.pdf]

Data Sharing Statement

Bernstein. Comparison of Electronic Health Record Messages to Mental Health Care Professionals Before vs After COVID-19 Pandemic. *JAMA Netw Open*. Published online July 24, 2023.
doi:10.1001/jamanetworkopen.2023.25202

Data
Data available: No
